# Odor-Based Recognition of Familiar and Related Conspecifics: A First Test Conducted on Captive Humboldt Penguins (*Spheniscus humboldti*)

**DOI:** 10.1371/journal.pone.0025002

**Published:** 2011-09-21

**Authors:** Heather R. Coffin, Jason V. Watters, Jill M. Mateo

**Affiliations:** 1 Institute for Mind and Biology, The University of Chicago, Chicago, Illinois, United States of America; 2 Committee on Evolutionary Biology, The University of Chicago, Chicago, Illinois, United States of America; 3 Chicago Zoological Society, Brookfield, Illinois, United States of America; Institut Pluridisciplinaire Hubert Curien, France

## Abstract

Studies of kin recognition in birds have largely focused on parent-offspring recognition using auditory or visual discrimination. Recent studies indicate that birds use odors during social and familial interactions and possibly for mate choice, suggesting olfactory cues may mediate kin recognition as well. Here, we show that Humboldt penguins (*Spheniscus humboldti*), a natally philopatric species with lifetime monogamy, discriminate between familiar and unfamiliar non-kin odors (using prior association) and between unfamiliar kin and non-kin odors (using phenotype matching). Penguins preferred familiar non-kin odors, which may be associated with the recognition of nest mates and colony mates and with locating burrows at night after foraging. In tests of kin recognition, penguins preferred unfamiliar non-kin odors. Penguins may have perceived non-kin odors as novel because they did not match the birds' recognition templates. Phenotype matching is likely the primary mechanism for kin recognition within the colony to avoid inbreeding. To our knowledge this is the first study to provide evidence of odor-based kin discrimination in a bird.

## Introduction

Mechanisms favoring inbreeding avoidance, such as kin recognition, should be important to species with natal-site philopatry [1]. Kin recognition is mediated by at least two mechanisms [2]. Through social interactions, animals can learn the phenotypes of related individuals during early development (e.g. parents, siblings), and later discriminate these familiar relatives from unfamiliar animals (‘prior association’). Second, animals can learn their own phenotypes and/or those of their familiar kin, and later compare or match the phenotypes of unknown animals to this learned template (‘phenotype matching’). Although both mechanisms involve a comparison between encountered phenotypes and recognition templates, prior association leads to recognition of previously encountered familiar individuals, whereas phenotype matching permits ‘recognition’ of unfamiliar kin, through generalization of learned recognition templates. Olfactory cues underlie kin recognition in many taxa [e.g. 3], [4], [5], yet most studies of kin recognition in birds have focused on the auditory or visual modalities [e.g. 6], [7]. For example, bank swallows use visual cues to recognize offspring, whereas colonial cliff swallow chicks produce signature calls to facilitate parent-offspring recognition [6]. Results of avian kin-recognition studies have been mixed, likely because of the highly varied social systems examined and methods used [see 8].

Until recently, olfactory abilities in birds have been regarded as weak or non-existent [9], [10]. However, across avian species the number of olfactory receptor genes (OR) is high regardless of ecological niche, and the total number of OR genes (rather than proportion of genes that are functional) correlates with olfactory bulb size and likely olfactory ability [11]. Recent studies of bird olfaction suggest that odors may be used in ecological, social and familial interactions [12], [13], [14], [15], [16], [17], including locating foraging patches, nest recognition, mate choice and social recognition. However, to date it is unknown whether olfaction mediates kin recognition in birds.

Procellariiform seabirds (petrels, albatrosses, and shearwaters) are known for their large olfactory bulbs and acute sense of smell, as they use odors for foraging and navigation. Odors are likely the dominant sensory cues used by chicks reared in burrows, and odor preferences may be learned through interactions with parents in the nest [13]. Odors may also play a role in social and familial interactions. Penguins (Sphenisciformes) and procellariiforms are phylogenetically related [18] and share several common features, including natal philopatry, large olfactory bulbs and olfactory acuity [14], [19]. Humboldt penguins (*Spheniscus humboldti*; Figure S1) are endangered [20], [21], long-lived monogamous birds, living in large colonies of closely spaced burrows on rocky mainland shores, especially near cliffs, or on islands off the coast [22], [23]. Both parents invest in offspring, and chicks fledge at about 10 to 12 weeks of age, leaving the breeding site to forage [possibly using olfactory cues; 24] along the coast for several months before returning to establish their own nests, typically within their natal colony [25]. Because of their natal philopatry, selection may favor kin recognition abilities in *S. humboldti* to avoid inbreeding.

Prior research has shown that seabird odors can be used to discriminate among mates and other conspecifics [17], [26], although it is unclear what odors are used. For example, the odors of blue petrels are individually discriminable by mice [27], but which particular odors are used for recognition remains unknown. Humboldt penguins, like many birds, preen themselves by distributing oil from the uropygial gland, or preen gland, to their feathers. The gland is especially well developed in aquatic birds, providing water-repellency and maintaining skin and plumage [28]. The composition of preen oil can differ between birds of different ages, sex, reproductive status, and diet [reviewed in 29]; [ see also 30], [31], and thus can possibly act as a recognition cue. Here we tested whether preen-gland odors can mediate social discrimination in captive Humboldt penguins.

## Materials and Methods

We conducted this research at Brookfield Zoo, Illinois, USA, in July and December of 2009. The 11 penguins housed in the public ‘Exhibit’ group comprised breeding pairs, their chicks, and other non-breeding individuals (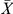 = 7.29 yr old + 1.38 SE, range 0.75–13 yr). The 11 unmated penguins housed ‘Off Exhibit’ comprised non-breeding individuals or hand-reared animals (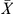 = 10.33 yr old + 2.79 SE, range 1.5–28 yr). Three individuals in the Off Exhibit group had been separated from Exhibit penguins for 4–12 years. Specimen reports were examined to create complete enclosure histories for each penguin. Familiarity and relatedness of individuals were determined from these reports.

Odors were collected by rubbing the preen gland with three Puritan cotton swabs (Hardwood Products Company, Guilford ME) 5 times by zookeepers wearing latex gloves. After removing most of the swabs' shafts each sample was placed in a 1.5 mL microcentrifuge tube and stored at −9° C until testing. We presented 9 Exhibit and 3 Off Exhibit birds (7 males, 5 females) with odors from a familiar non-kin and an unfamiliar non-kin or odors from an unfamiliar kin (coefficient of relationship, *r*, = 0.125–0.5) and an unfamiliar non-kin, with 10 birds experiencing both tests (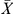 = 77.4 days in between tests + 27.94 SE; all but one bird had the kin-nonkin test first).

Preference tests were conducted in a separate holding room (∼3 m×4.5 m) that contained only two medium-sized dog kennels. Penguins here use kennels as nests and spend a significant amount of time inside them, and thus we used them to present odors. Six identical kennels were used and two were selected arbitrarily from the six for each test. Kennels were centered in the room, 1 m apart, facing the door which had a small window allowing observation from the hallway. For each test, a zookeeper thawed two odor samples for 3 minutes, rubbed one sample around the interior walls and ceiling of the first kennel, and then rubbed it onto a coffee filter which was placed under a mat on the bottom of the kennel. This procedure was repeated in the second kennel with the second odor, so that the odors were presented simultaneously. Donors for each pair of odors were the same sex. Placement of kennels and odors was arbitrary across tests. The person recording behavioral data was blind to the placement of the odors and the identity of the odor donors.

Penguins were free to explore the room and the kennels for 10 to 15 minutes (durations differed due to zookeeper duties), although only data from the first ten minutes of each test were used for analyses. We recorded with a stopwatch time investigating (head within 15.2 cm of kennel door or inside the opening), time inside each kennel, and latency to enter each kennel. We also recorded the number of investigations per kennel, the number of entries per kennel, which kennel birds investigated first, and which kennel birds entered first. Kennels were cleaned with a bleach and water solution after each test to eliminate odors. The solution was the same as that used daily by zookeepers to clean and eliminate odors from kennels and holding rooms. All tests were conducted between 1200 and 1430 hr. Our research followed the ABS/ASAB *Guidelines for the Use of Animals in Research*, and was approved by Brookfield Zoo's Biological Research Steering Committee (#255), and adheres to standards set forth by the NIH for animal research.

We used two-tailed paired *t*-tests on log-transformed data or nonparametric Wilcoxon tests when data were not normally distributed (verified with Kolmogorov-Smirnov tests) in SPSS (v. 12) to evaluate odor discrimination. Two-tailed binomial tests determined if there were differences in which kennel birds investigated and entered first. Despite the limitation in the number of penguins that could be tested, the statistics we used are robust enough to detect discrimination between odor types.

## Results

### Familiar vs. unfamiliar non-kin odors

**Figure 1:**
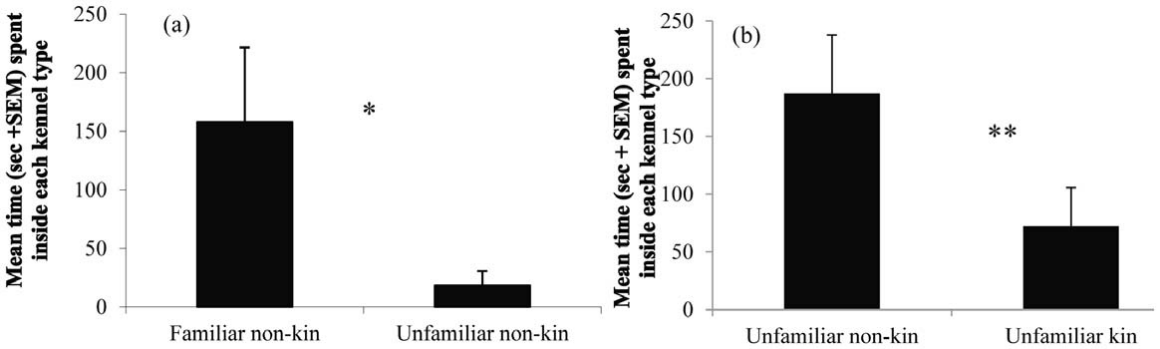
Mean time (sec + SEM) inside kennels containing preen-gland odors. (a) Odors from familiar and unfamiliar non-kin (*n* = 10). (b) Odors from unfamiliar kin and non-kin (n = 12). * P<0.05 **P<0.01.

Penguins first investigated kennels with odors from unfamiliar non-kin before kennels with odors from familiar non-kin (7 of 8 birds, binomial distribution, *P* = 0.07). Two other birds did not investigate either kennel. Penguins spent more time inside kennels with familiar odors than inside kennels with unfamiliar odors (Wilcoxon signed-rank test; *Z* = 1.992, *P* = 0.046). Total time investigating and inside kennels was longer for familiar kennels than unfamiliar kennels (*Z* = 1.836, *P* = 0.066; Figure 1a).

### Unfamiliar kin vs. unfamiliar non-kin odors

**Figure 2:**
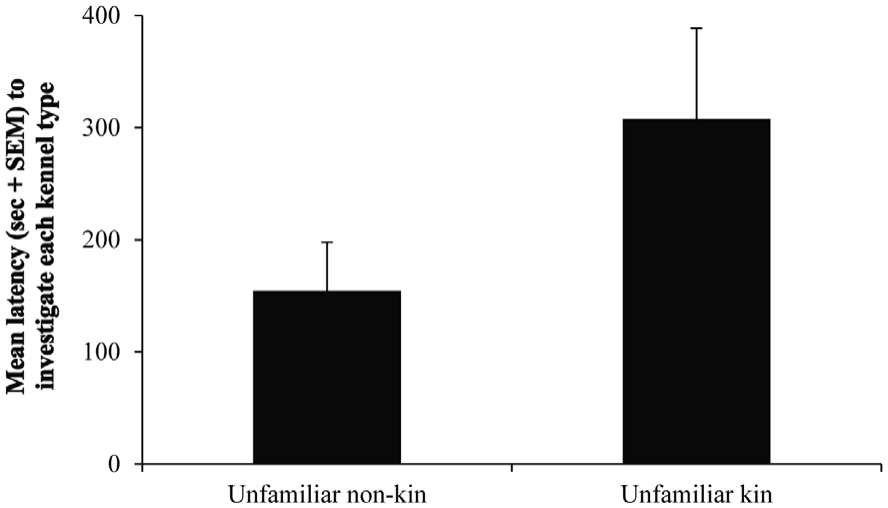
Mean latency (sec + SEM) to investigate (head within 15.2 cm of kennel opening) kennels containing preen-gland odors of unfamiliar kin and non-kin (*n* = 12).

Penguins were more likely to first enter kennels containing odors of unfamiliar non-kin than odors of unfamiliar kin (9 of 12 birds, binomial distribution, *P* = 0.146). The latency to investigate non-kin odors was shorter than it was for kin odors (*Z* = 1.784, *P* = 0.074; Figure 2). The latency to enter kennels with non-kin odors was shorter than for kin odors (*Z* = 1.647, *P* = 0.099). Penguins spent more time inside kennels containing non-kin odors than kin odors (log-transformed data; *t*_11_ = 3.434, *P* = 0.006; Figure 1b), and total time investigating and inside kennels was longer for non-kin odors than kin odors (*t*_11_ = 2.724, *P* = 0.02). For all tests, no effects of sex or mating status (previously mated or not) were found.

## Discussion

The results demonstrate that captive Humboldt penguins use olfactory cues to recognize and discriminate between familiar and unfamiliar individuals and between kin and non-kin. Our preference tasks provided a simultaneous choice of two odors, and birds differentially investigated and entered the kennels in the absence of any visual or auditory cues. In the first experiment, birds recognized the odors of familiar unrelated individuals through the mechanism of prior association. In the second experiment, birds discriminated unfamiliar kin odors from unfamiliar non-kin odors through the mechanism of phenotype matching.

We found that Humboldt penguins investigated the kennels with unfamiliar odors first but then spent more time inside kennels with familiar odors (Fig. 1a). This indicates that Humboldt penguins were initially interested in unfamiliar or novel odors as demonstrated in studies of other vertebrate species [e.g. 3], [32], but afterward familiar odors were preferred. Prior association would allow recognition of nest-mates or colony mates, but it would not permit recognition of siblings from different breeding seasons.

In our tests of kin recognition, penguins had shorter latencies to investigate and enter kennels with odors of unfamiliar non-kin compared with unfamiliar kin, and spent more time investigating and inside kennels containing non-kin odors (Figs. 1b; 2). Although not all results reached significance at α = 0.05, they trended in the expected direction. With the phenotype-matching mechanism, animals develop a template of kin traits (e.g., odors) and later match the phenotypes of an unfamiliar individual to this template; the degree of match to the template indicates the degree of relatedness between the two. Here, non-kin odors would not match birds' recognition templates as well as kin odors, and therefore as novel odors they would be investigated quicker. More importantly, birds spent significantly more time inside kennels with non-kin odors (Fig. 1b), which least matched their recognition templates, further demonstrating an ability to discriminate odors based on genetic differences.

Most studies of bird olfaction explain self-odor and mate-odor recognition as a homing mechanism. Preference for kin and familiar odors may be associated with locating burrows after returning from foraging at night (reviewed in [13]). In Humboldt penguins, breeding pairs take turns foraging at sea during the chick-rearing period [33], thus favoring discrimination of familiar (e.g. mate, nest) and unfamiliar odors upon return to the colony. They also exhibit mate and nest-site fidelity [22], [23]. Therefore full siblings will be born in different years and due to natal philopatry [25] may encounter each other. Phenotype-matching abilities allow birds to discriminate among conspecifics as a function of relatedness, and thus avoid mating with unfamiliar siblings.

Although based on a small sample of captive individuals, our study is the first to demonstrate odor-based kin discrimination in a bird, and future work will explore how odor discriminations influence *S. humboldti* social relationships. Such continued research will provide useful information for conservation workers and increase the success of captive breeding programs for this endangered species. Knowledge of the types and extent of social recognition is critical for the design of captive-breeding programs or for the release of endangered species into the wild, particularly if familiarity influences the formation and stability of social groups or their mating success [34]. This study underscores our lack of understanding of the chemosensory world of birds, but we anticipate it will stimulate investigations of olfactory abilities of a wide range of avian species across a wide range of contexts.

## Supporting Information

Figure S1 Penguin and chick. Photo credit is to Jim Schultz, Chicago Zoological Society.
(JPG)
