# Supplementary figures and images for: Odor-Based Recognition of Familiar and Related Conspecifics: A First Test Conducted on Captive Humboldt Penguins (Spheniscus humboldti)
Source: PLoS One. 2011 Sep 21;6(9):e25002. doi: 10.1371/journal.pone.0025002 (PMC3177858; doi:10.1371/journal.pone.0025002)

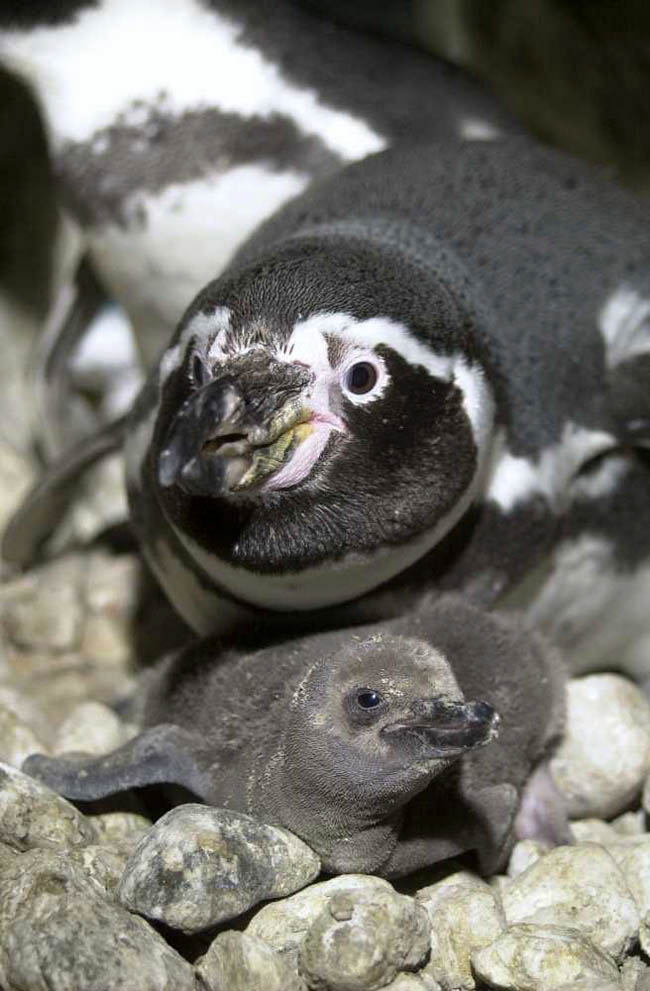

Supplement: Figure S1 — Penguin and chick. Photo credit is to Jim Schultz, Chicago Zoological Society. (JPG) [file pone.0025002.s001.jpg]
